# Identification and validation of tolerogenic dendritic cells-related biomarkers in diabetic retinopathy

**DOI:** 10.1007/s13340-026-00914-5

**Published:** 2026-07-05

**Authors:** Chun-yi Wei, Song-man Li, Ling-juan Liu, Jun-peng Huang, Xiao-hui Lai, Li-juan Yang, Wen-wang Liang

**Affiliations:** Department of Ophthalmology, The Second Affiliated Hospital of Guangxi University of Chinese Medicine, Nanning, 530011 Guangxi China

**Keywords:** Diabetic retinopathy, Tolerogenic dendritic cells, Biomarkers, TFEC, CHMP2A

## Abstract

**Background:**

Diabetic retinopathy (DR) is a primary microvascular complication of diabetes. Its pathogenesis is associated with chronic inflammation and immune responses. While tolerogenic dendritic cells (tolDCs) are critical for suppressing excessive inflammation and maintaining immune homeostasis, their function in DR is not well characterized.

**Objective:**

To identify biomarkers associated with tolDCs in DR and to explore their mechanisms.

**Methods:**

Biomarkers were identified from public databases using differential analysis, machine learning (LASSO regression analysis and Random Forest analysis), and expression validation. Their mechanisms were then explored through enrichment analysis and immune infiltration, and ultimately validated by reverse transcription-quantitative PCR (RT-qPCR) on clinical blood samples.

**Results:**

A total of 2096 differentially expressed genes (DEGs)1 were identified between DR and control groups. By intersecting with 6267 tolDCs-related genes, 51 key candidates were selected. Machine learning and expression validation further pinpointed two biomarkers: transcription factor EC (TFEC) and chromatin modifying protein 2 A (CHMP2A). Gene Set Enrichment Analysis (GSEA) showed significant enrichment of TFEC in 994 pathways and CHMP2A in 748, with co-enriched pathways including apoptosis, VEGFA-VEGFR2 signaling, and VEGF signaling. Analysis identified eight altered immune cell types; TFEC correlated strongest with activated NK cells (cor = − 0.49, *p* < 0.001), while CHMP2A correlated with resting mast cells (cor = 0.49, *p* < 0.001). RT-qPCR confirmed TFEC upregulation and CHMP2A downregulation in DR, validating the bioinformatic results.

**Conclusion:**

TFEC and CHMP2A are potential biomarkers involved in DR pathogenesis via tolDCs.

## Introduction

Diabetic retinopathy (DR) is a leading blinding complication of diabetes mellitus, affecting 30–40% of diabetic patients and *causing* visual impairment or blindness in over 100 million people globally [1]. Diabetic macular edema is the *predominant* cause of central vision loss in DR patients. As an immune-privileged tissue, the retina maintains strict homeostasis to resist systemic immune aggression. However, long-term hyperglycemia-induced oxidative stress and microangiopathy disrupt blood-retinal barrier integrity, triggering macular edema, microvascular leakage and retinal neurodegeneration, which constitute the core pathological mechanism of DR [2–3].

Substantial evidence indicates that chronic inflammation and immune dysregulation are closely associated with the pathogenesis of DR. DR is widely recognized as a chronic low-grade inflammatory disorder, termed retinal microinflammation. Inflammatory activation persists throughout DR progression, characterized by elevated systemic and intraocular inflammatory mediators, including increased serum C-reactive protein, peripheral neutrophils and intraocular inflammatory biomarkers [4–5] . In DR models and clinical specimens, retinal endothelial cells upregulate leukocyte adhesion molecules, leading to leukostasis in microcirculation and aggravated local immune infiltration [6]. Sustained low-grade inflammation further damages retinal neurons and vascular endothelium, induces chronic maculopathy, and accelerates DR deterioration [2]. Currently, intravitreal anti-vascular endothelial growth factor (anti-VEGF) agents and long-acting hormones are primary treatments to inhibit or delay DR progression. However, a significant proportion of patients exhibit poor or no response to these intravitreal therapies, ultimately progressing to irreversible blindness [7]. Therefore, clarifying DR core pathogenesis and identifying reliable early biomarkers and novel therapeutic targets are urgently required for current DR research. In recent years, numerous studies have explored inflammatory biomarkers as objective indicators to evaluate the relationship between systemic inflammation and diabetic complications [8]. The systemic immune-inflammation index serves as a sensitive indicator to reflect host immune and inflammatory status, with great potential for early prediction and individualized management of diabetic complications [9].

 Tolerogenic dendritic cells (tolDCs) are a semi-mature dendritic cells (DCs) subset with low immunogenicity and strong immunomodulatory functions, existing in peripheral tissues or induced in vitro. TolDCs secrete some cytokines such as IL-10 and TGF-β, remodel immune microenvironment, and drive immunosuppressive cell differentiation to maintain immune homeostasis [10–12]. Together with regulatory T cells (Tregs), tolDCs form a critical immune tolerance axis. TolDCs induce Tregs differentiation via low expression of co-stimulatory molecules (CD80/CD86) and stabilize Tregs immunosuppressive function. In turn, Tregs inhibit DCs co-stimulatory molecules through cytotoxic T-lymphocyte-associated antigen-4 (CTLA-4) to consolidate tolDCs phenotypes. This bidirectional tolDCs-Tregs feedback loop restrains excessive inflammation and maintains immune homeostasis [13–14].

 In diabetes, persistent hyperglycemia and oxidative stress alter tolDCs phenotypes and functions, switching them from a tolerogenic to a pro-inflammatory state and disrupting the tolDCs/Tregs axis. This functional abnormality impairs tolDCs’ ability to induce and maintain Tregs function, reducing regulatory B cell function, sustaining NF-κB and other inflammatory pathway activation, and exacerbating immune disorder, vascular damage [15]. Studies have shown that diabetic corneal nerve injury occurs before retinal microvascular damage, and the reduction of immature tolDCs accompanied by increased mature DCs in the cornea may serve as early biomarker for DR [16]. DR is closely associated with dysfunction of tolDCs. Abnormally activated mature DCs exacerbate retinal inflammation. NECA, an adenosine receptor agonist, inhibits DCs maturation and the TLR4-MyD88-NF-κB pathway, enhances anti-inflammatory responses, and restores immune homeostasis to ameliorate DR [17]. TolDCs inhibit the development of autoimmune-mediated hyperglycemia into insulin-dependent type 1 diabetes mellitus, while preserving a critical mass of β cells capable of restoring normal blood glucose levels to some extent in newly diagnosed patients [18]. Clinical studies have shown that subcutaneous injection of tolDCs can effectively suppress antigen-specific pro-inflammatory T cells in patients with type 1 diabetes, leading to the generation of Tregs and IL-10, thereby intervening in the progression of the disease [19]. Despite encouraging preclinical evidence, the clinical translation of tolDCs is hindered by unstable cell phenotypes, poor in vivo stability and undefined administration routes. Even so, key molecules governing the tolDCs-mediated immune tolerance axis provide novel targets for DR early warning and intervention.

This study focused on tolDCs, combining bioinformatics analysis, immune infiltration evaluation and clinical validation to screen core biomarkers closely related to tolDCs function in DR. We further analyzed their expression profiles, immune correlations and clinical implications during DR progression. This research aims to reveal the mechanism of tolDCs-mediated immune tolerance imbalance in DR, identify non-invasive peripheral blood biomarkers for early DR monitoring, and provide *a* theoretical and experimental basis for novel immunotherapies targeting immune tolerance restoration.

## Materials and methods

### Data acquisition

**Fig. 1:**
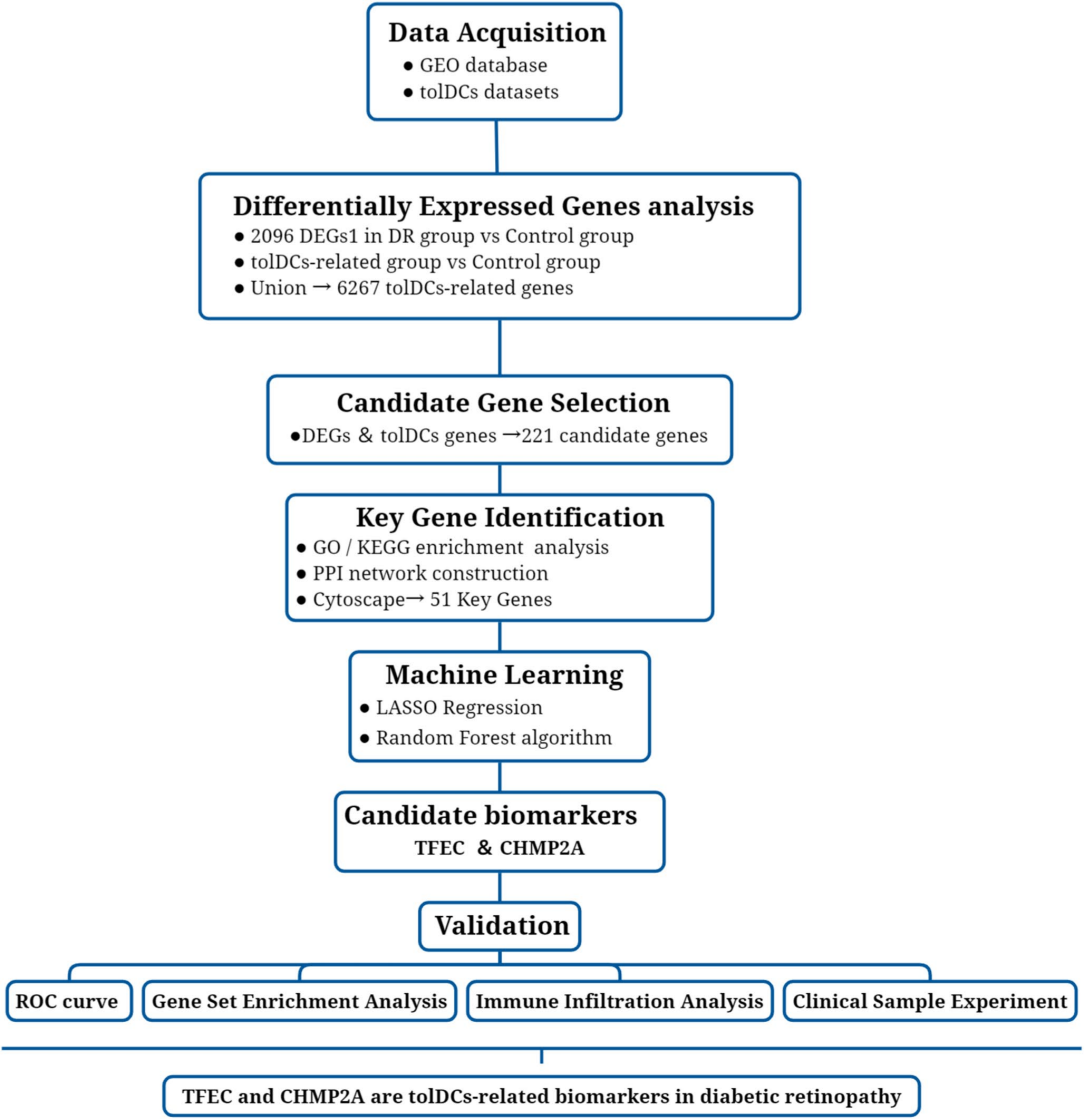
Schematic overview of the bioinformatics workflow

All gene expression data analyzed in this DR investigation were obtained from the GEO database (Accessed on February 28th, 2025). The GSE221521 dataset (platform GPL24676) was designated as the training cohort, comprising 69 DR patient peripheral blood samples and 50 control samples, with other available samples excluded from analysis. The GSE185011 (GPL24676) dataset was used as the validation set, the dataset comprised matched cohorts of 5 DR patients and 5 healthy controls, all analyzed using peripheral blood mononuclear cells (PBMCs), with the other samples excluded. The GSE23371 (GPL570), GSE52894 (GPL10558), GSE182528 (GPL570), and GSE56017 datasets were used as datasets related to tolDCs. In the GSE23371 dataset, 3 activated tolerogenic dendritic cell samples (tolDCs group) and 3 tolerogenic precursor dendritic cell samples (control group) were selected, with the remaining samples excluded. In the GSE52894 dataset, 5 lipopolysaccharide-treated tolDCs samples (tolDCs group) and 4 tolerogenic precursor dendritic cell samples (control group) were selected, with the other samples excluded. In the GSE182528 dataset, 5 tolDCs samples derived from healthy donor in vitro (tolDCs group) and 5 monocyte-derived dendritic cell samples were selected (control group), with the remaining samples excluded. In the GSE56017 dataset, 8 tolerogenic monocyte-derived dendritic cell samples (tolDCs group) and 7 mature monocyte-derived dendritic cell samples (control group) were selected, with the other samples excluded. A schematic overview of the entire bioinformatics workflow is provided in Fig. 1.

**Fig. 2:**
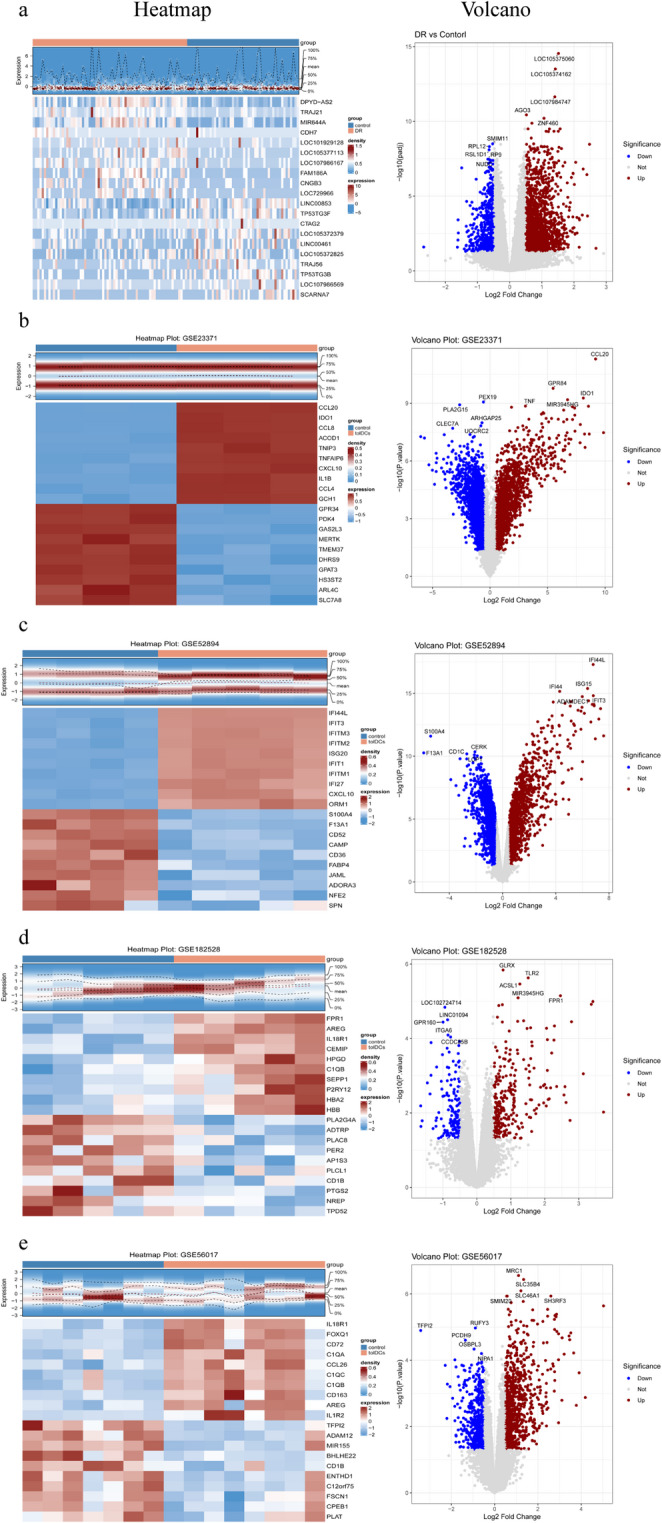
DEGs Profiles in DR and tolDCs from GEO Datasets. Panels **a–e** show DEGs profiles from datasets GSE221521 (DR), GSE23371 (tolDCs), GSE52894 (tolDCs), GSE182528 (tolDCs), and GSE56017 (tolDCs), respectively. Total 6,267 tolDCs-related genes were identified. Left: Heatmap=DEGs expression; rows: genes; columns: samples. Right: Volcano plot: red/blue = significant up/downregulated DEGs (|log_2_FC|>1, adjusted *p* < 0.05)

**Fig. 3:**
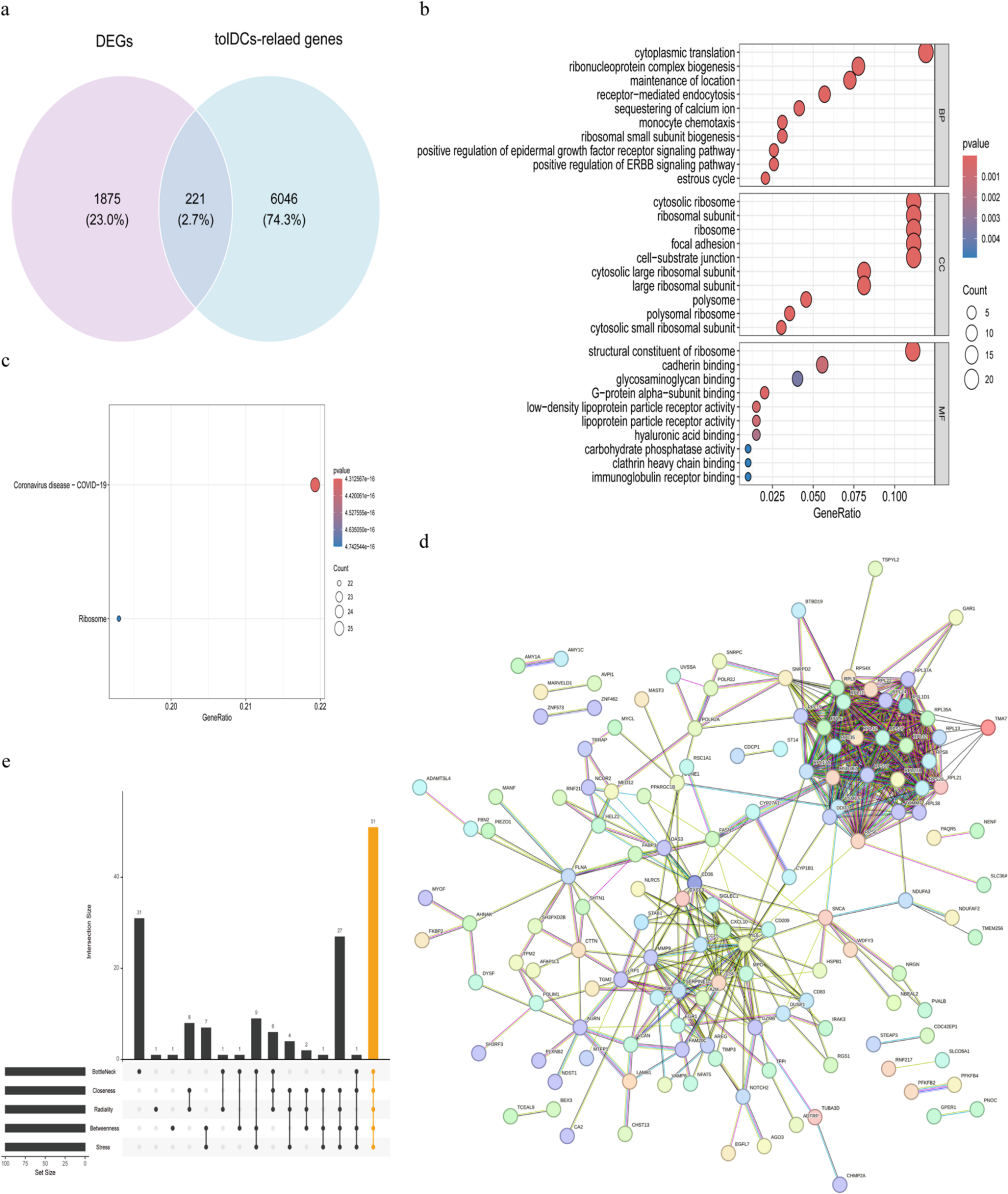
Identification and functional analysis of tolDCs-associated candidate genes. **a** Venn diagram illustrating the overlap between DEGs and tolDCs-related genes, identifying 221 overlapping candidate genes. **b** GO enrichment analysis of the candidate genes, showing significant terms in biological process, cellular component, and molecular function categories. **c** KEGG pathway enrichment analysis highlighting the top enriched pathways, including “Coronavirus disease-COVID-19” and “Ribosome.” **d** PPI network of candidate genes, displaying interactions among 203 proteins such as OSA3 and FASN. **e** Final selection of 51 key genes using five algorithmic criteria

**Fig. 4:**
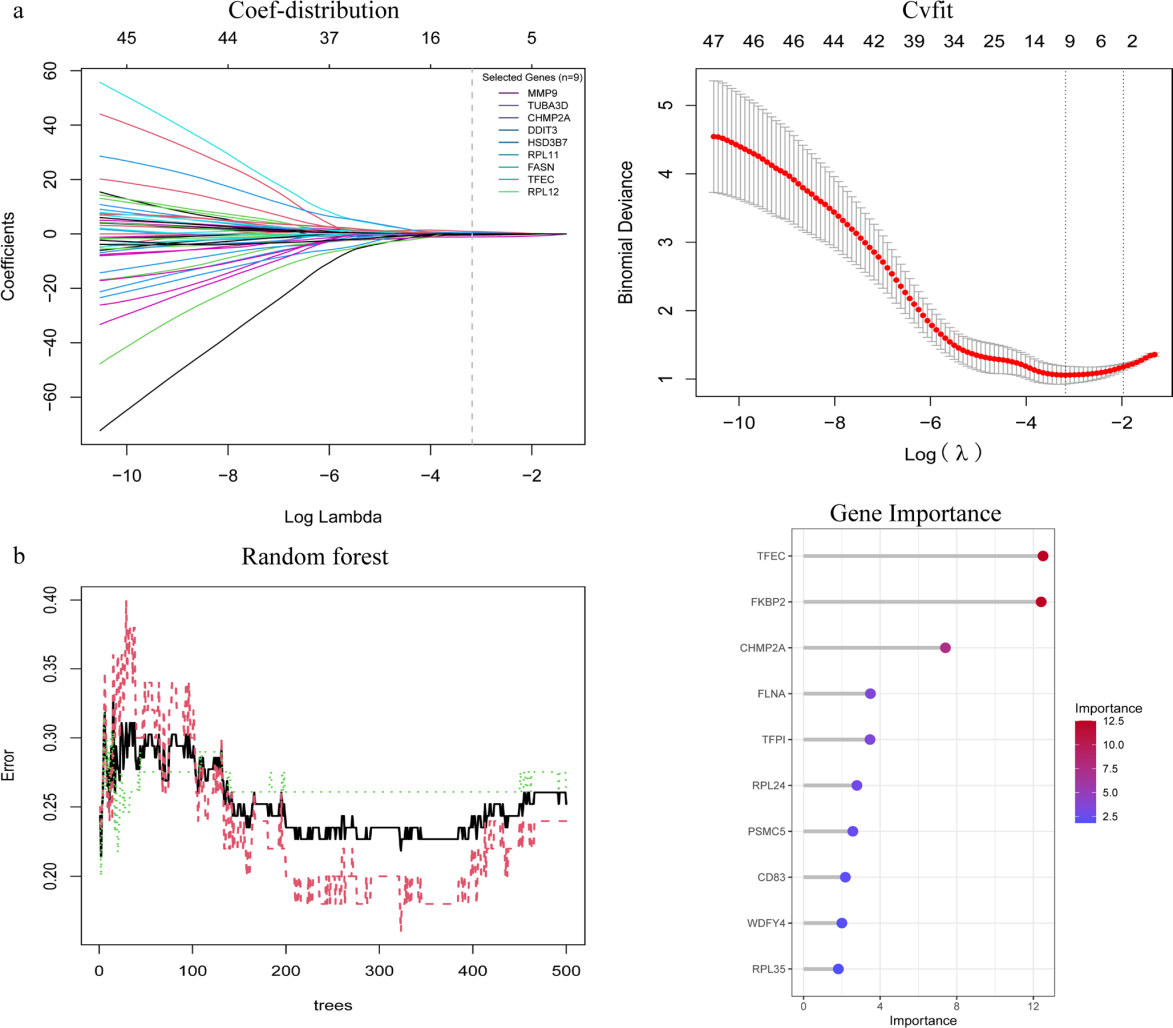

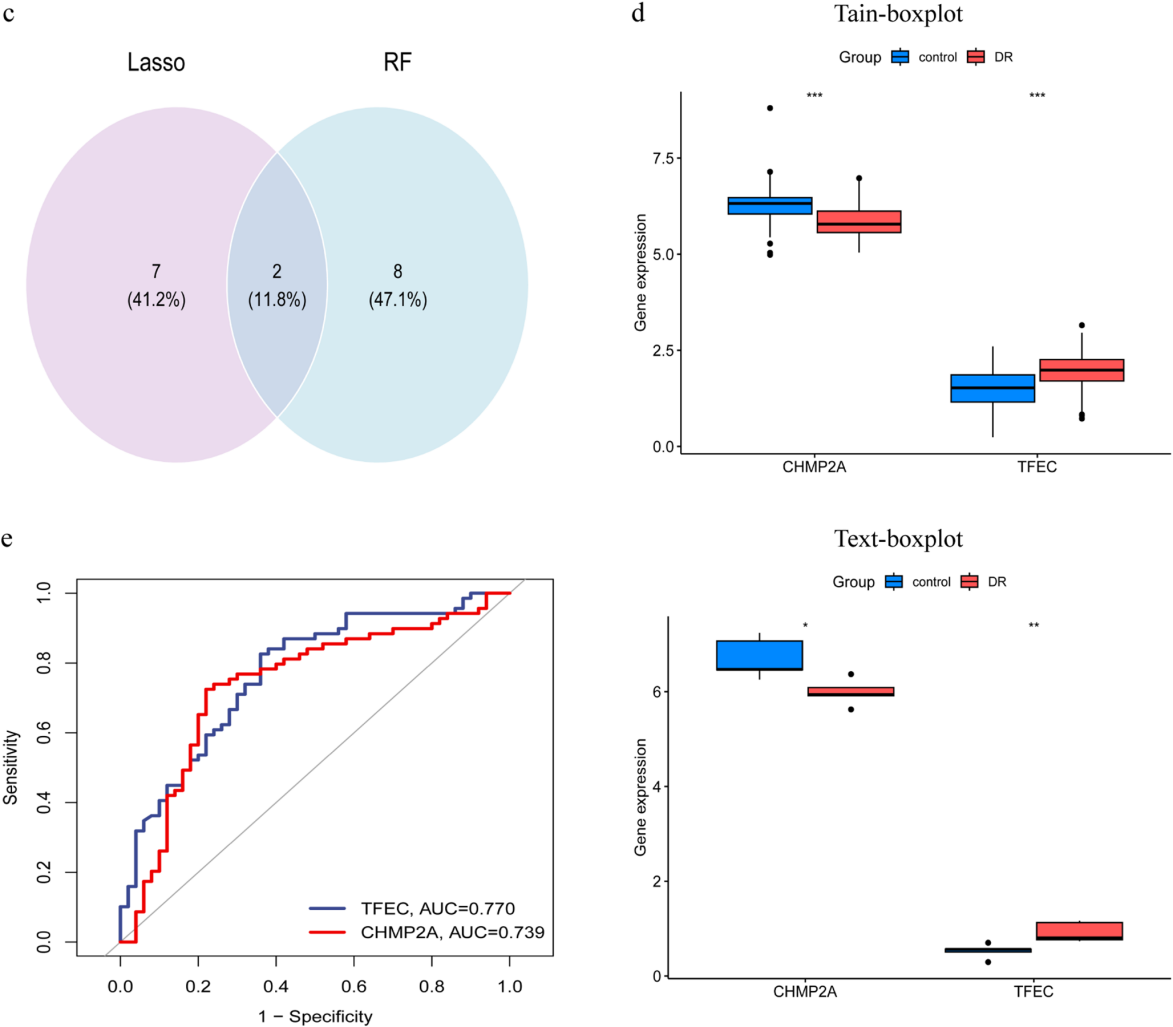
Machine learning-based biomarker discovery and validation for DR. **a** LASSO regression identified 9 feature genes (lambda.min = 0.04). **b** Random Forest ranked the importance of the top 10 feature genes. **c** Venn diagram reveals TFEC and CHMP2A as common biomarkers from both methods. **d** Validation confirms differential expression of TFEC and CHMP2A (*p* < 0.05). **e** ROC curves demonstrate the diagnostic value of TFEC (AUC = 0.770) and CHMP2A (AUC = 0.739)

**Fig. 5:**
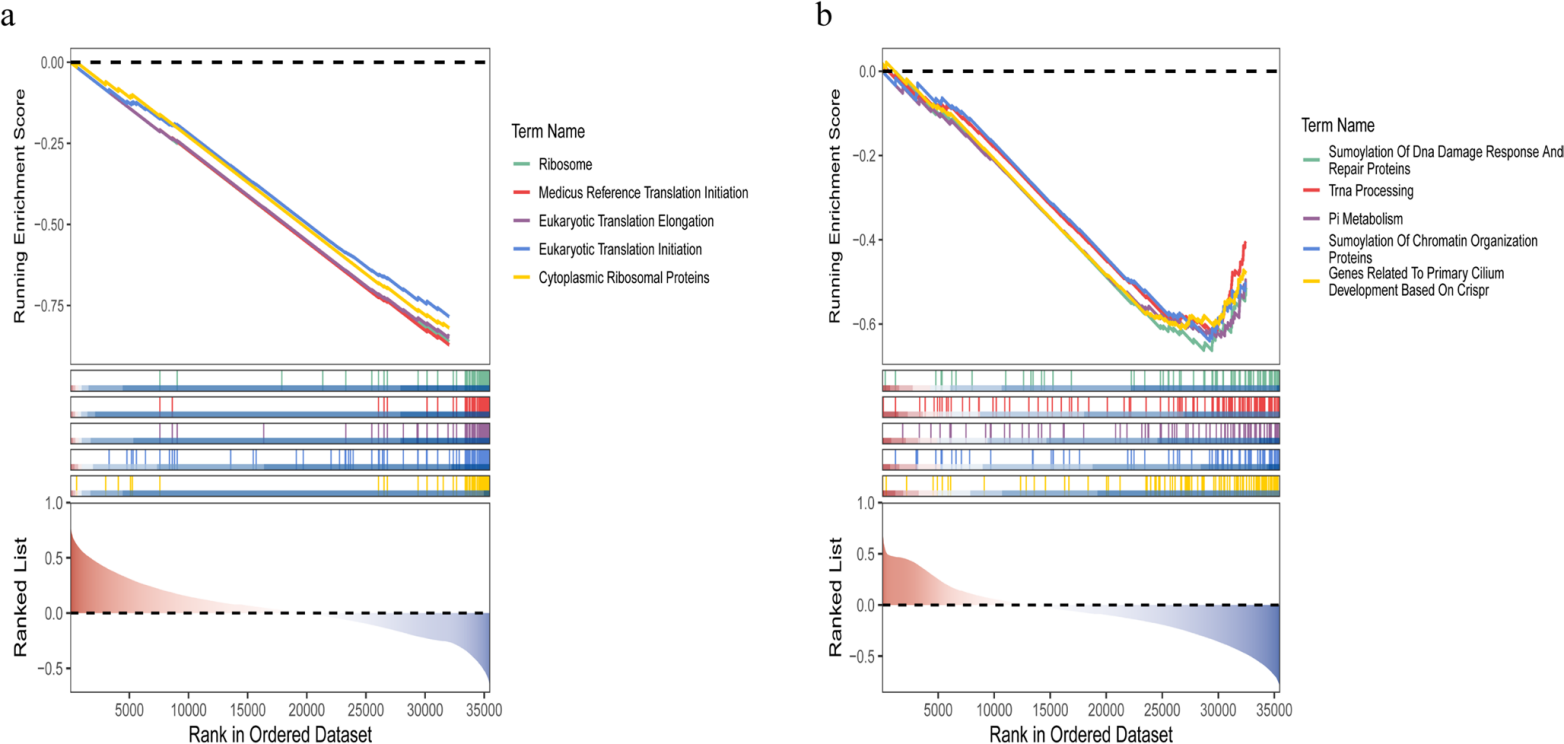
Gene Set Enrichment Analysis (GSEA) of TFEC and CHMP2A in DR. a: GSEA plot showing significant enrichment of TFEC-associated genes in the “Cytoplasmic Ribosomal Proteins” pathway. b: GSEA plot indicating enrichment of CHMP2A-associated genes in the “tRNA Processing” pathway Shared enriched pathways for both genes include apoptosis, VEGFA-VEGFR2 signaling, and VEGF-mediated signaling

**Fig. 6:**
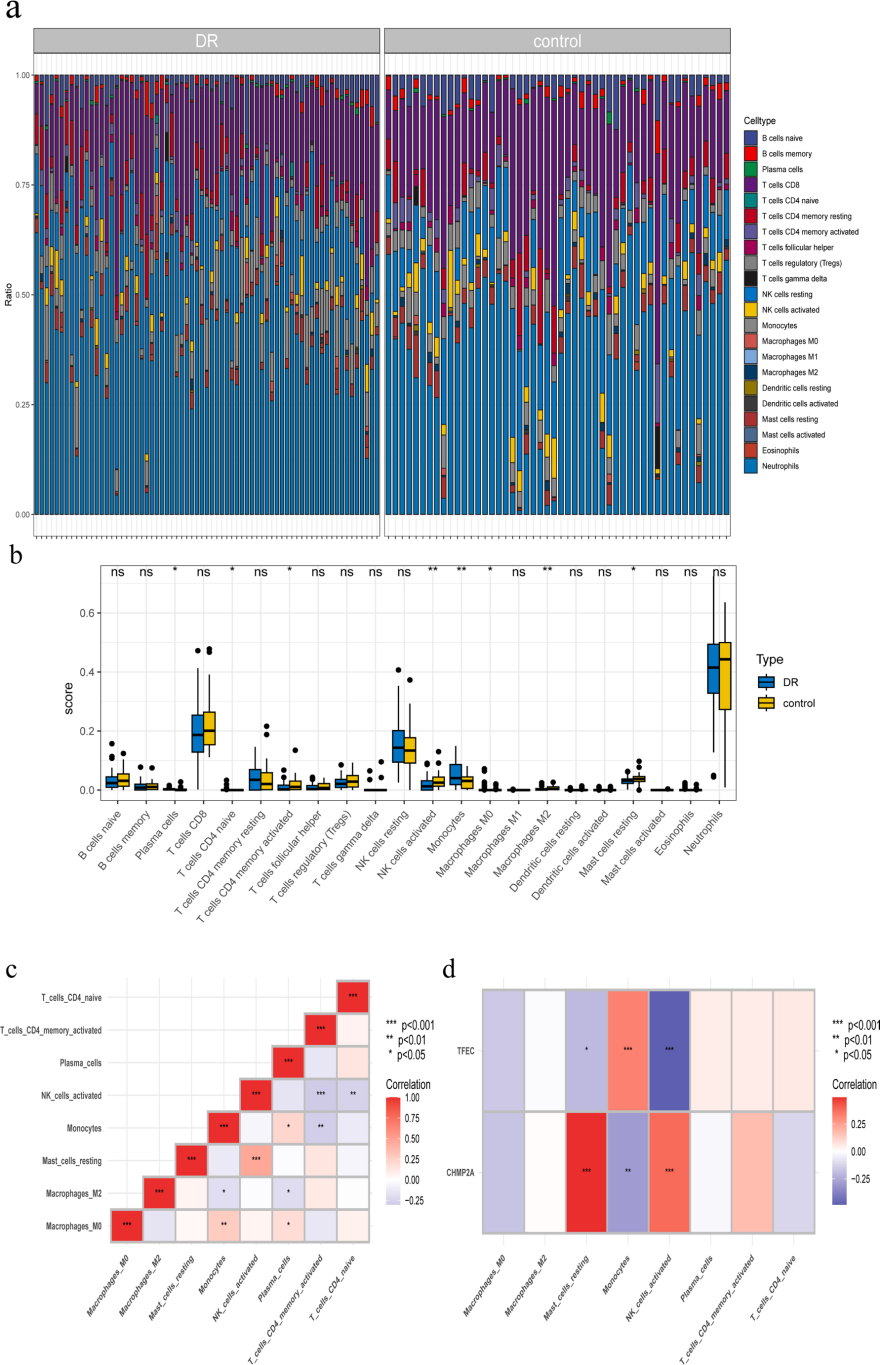
Analysis of immune infiltration and biomarker correlation in DR. **a** Stacked bar plot showing the proportion of 22 immune cell types in DR and control groups. **b** Box plot showing immune cell scores, highlighting 8 cell types with significant differences between DR and control groups (*p* < 0.01). **c** Spearman correlation heatmap of immune cell types (strong NK/mast cell correlation shown; *p* < 0.001). **d** Biomarker-immune cell correlation heatmap (TFEC: negative with activated NK cells; CHMP2A: positive with resting mast cells; *p* < 0.001)

**Fig. 7:**
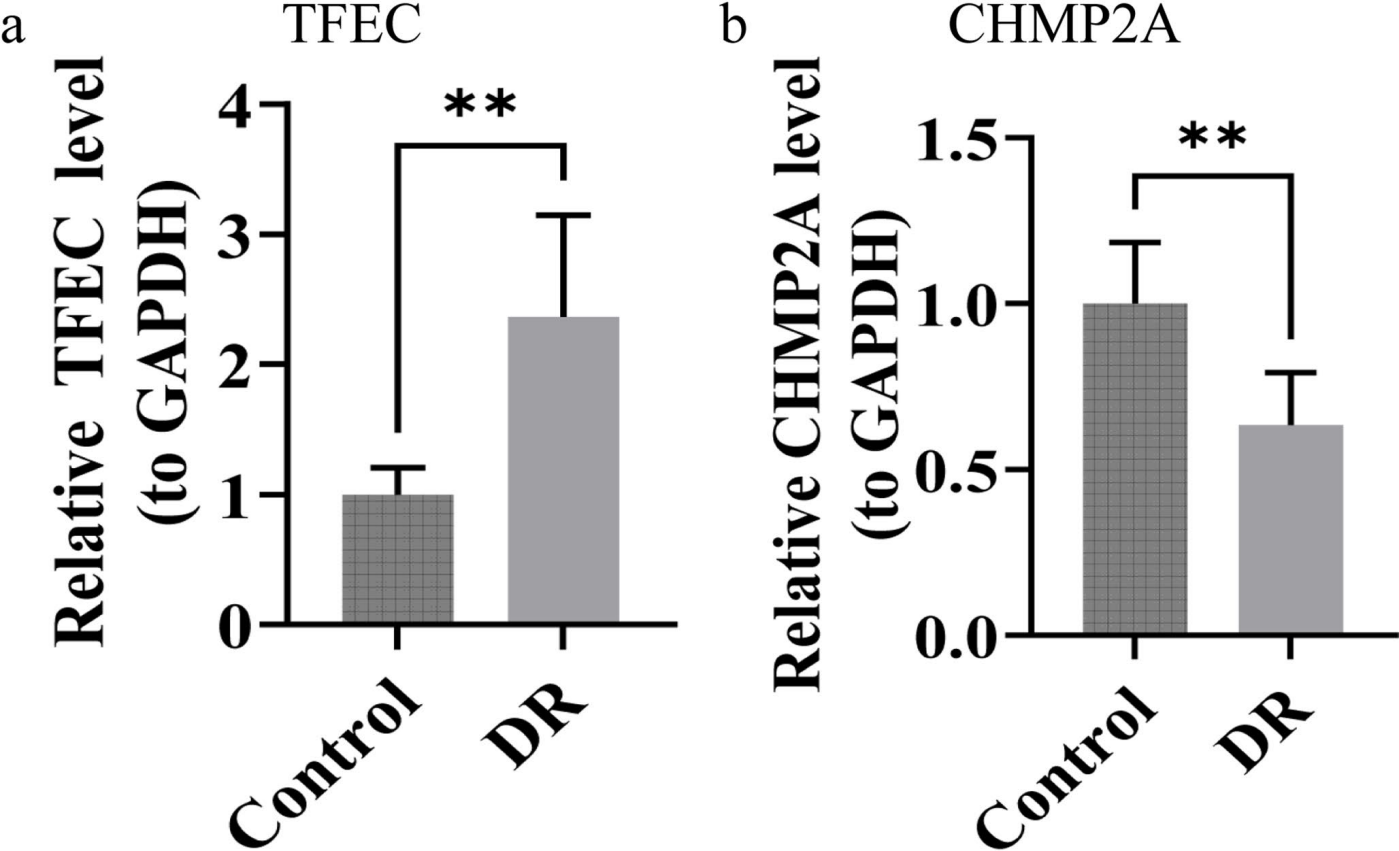
RT-qPCR validation of TFEC and CHMP2A expression in clinical samples. RT-qPCR confirmed TFEC upregulation and CHMP2A downregulation in DR (*p* < 0.01). This validates the bioinformatic findings

## DEGs analysis

The DESeq2 package (v 1.38.0) [20] was utilized to identify differentially expressed genes (DEGs)1 between the DR and control groups in the GSE221521 dataset (adj. *p* < 0.05 and |log_2_ FC| >0.5). The ggplot2 package (v3.4.1) was used to visualize DEGs1 through a volcano plot [21], annotating it to highlight the top five most significantly upregulated and downregulated genes. The ComplexHeatmap package (v 2.15.1) [22] was employed to construct a heatmap representation of the 10 *top* DEGs in both directions. Differential analysis between tolDCs and control groups was conducted on the GSE23371, GSE52894, GSE182528, and GSE56017 datasets using the limma package (v3.56.2) [23] (*p* < 0.05 and |log_2_ FC| >0.5). The methods for generating the volcano plot and heatmap were the same as described above. The resulting genes were respectively named DEGs2, DEGs3, DEGs4, and DEGs5. Subsequently, DEGs2, DEGs3, DEGs4, and DEGs5 were merged, and duplicate genes were removed to obtain the tolDCs-related genes.

### Key genes acquisition

The ggVenn package (v 0.1.10) [24] was used to integrate the DEGs1 with tolDCs-related genes. The obtained genes were designated as candidate genes. Based on candidate genes, the clusterProfiler package (v4.2.2) was employed to conduct comprehensive functional enrichment analysis of the gene sets [25], including GO (*p* < 0.05) and KEGG enrichments (*p* < 0.05). The results displayed the top 10 enriched terms and top 5 KEGG pathways, selected based on their respective gene counts. We generated a PPI network for candidate genes through STRING (Search Tool for Retrieval of Interacting Genes/Proteins), retaining only high-confidence interactions (interaction score > 0.15). The result was visualized using Cytoscape (v 3.10.2) [26]. Subsequently, the candidate genes were analyzed using five algorithms from CytoHubba: BottleNeck, Closeness, Radiality, Betweenness, and Stress. The consensus among the top 100 genes across the five algorithms was graphically represented using an upset plot visualization. The resulting genes were designated as key genes.

### Biomarkers acquisition

The glmnet package (v4.1.8) was employed to conduct LASSO regression analysis with optimal lambda selection [27] on key genes across all samples in the GSE221521 dataset, where genes that were not penalised to 0 were considered as feature genes 1. Additionally, the Random Forest package (v 4.7.1.2) [28] was used to perform the Random Forest algorithm. When the 5-fold cross-validation error rate reached its minimum, the importance of each gene was recorded. The most biologically significant genes were identified as feature genes based on their importance scores. Candidate biomarkers were identified by finding the overlapping genes between feature sets 1 and 2, with results displayed via ggVennDiagram (v0.1.10). Differential expression of candidate biomarkers was assessed using Wilcoxon rank-sum tests (*p* < 0.05) in both GSE221521 and GSE185011 datasets. Only genes showing consistent expression patterns (up/down-regulation) in both cohorts were ultimately validated as biomarkers. Additionally, a ROC curve was generated for the GSE221521 dataset using the pROC package (v1.18.5) [29], and the AUC value was calculated to evaluate the predictive performance of the biomarkers (AUC > 0.7).

### Gene set enrichment analysis (GSEA)

The reference gene set was obtained from the “c2.cp.v2024.1.Hs.symbols.gmt” file in MSigDB. Pathway enrichment analysis was conducted using the clusterProfiler package (v4.2.2) through GSEA methodology. The psych R package (v2.2.9) was employed to calculate comprehensive correlation matrices comparing each biomarker’s expression profile against all genes in GSE221521 [30]. The resulting correlation coefficients were ranked and these ranked lists were subjected to thresholding at |NES| > 1 and *p* < 0.05 to generate biomarker-associated gene lists. The top 5 pathways were presented based on *P*-value.

### Immune microenvironment analysis

The Wilcoxon rank-sum test (*p* < 0.05) was employed to identify statistically significant variations in immune cell proportions between DR patients and controls. Spearman correlation analysis was performed on all GSE221521 samples to explore the relationships between biomarkers and differential immune cell types, as well as between the immune cell types themselves, using the psych package (v 2.2.9) (|cor| > 0.30, *p* < 0.05).

## RT-qPCR

As a systemic microvascular complication accompanied by immune and inflammatory disorders, DR can be adequately assessed using peripheral blood. Peripheral blood is easily accessible, minimally invasive, and repeatable, making it ideal for clinical screening and follow-up. Conversely, retinal, vitreous, and aqueous humor samples require invasive collection with surgical risks and are unsuitable for routine or early detection. Accordingly, peripheral blood was used for experimental validation in this study.

This study enrolled a total of 10 volunteers, comprising 5 DR patients and 5 matched controls, who were recruited by the same attending physician at our hospital in January 2025. Peripheral blood samples were collected from all participants, and the expression levels of tolDCs-related biomarkers were experimentally validated using reverse transcription-quantitative polymerase chain reaction (RT-qPCR).

The inclusion criteria for the DR experimental group were as follows: (1) diagnosis of type 2 diabetes mellitus; (2) confirmation of DR by fundus photography, optical coherence tomography (OCT), or fluorescein angiography; (3) age between 45 and 85 years. The exclusion criteria for all participants were: (1) presence of severe systemic diseases (e.g., renal failure with an estimated glomerular filtration rate (eGFR) < 60mL/min/1.73 m², malignancy, or active immune diseases); (2) congenital or hereditary ocular diseases (e.g., congenital cataract, retinitis pigmentosa, or congenital/developmental glaucoma); (3) acute or progressive infectious eye diseases (e.g., infectious endophthalmitis or keratitis); (4) history of traumatic ocular diseases (e.g., traumatic cataract or ocular rupture); (5) history of ocular surgery within the past 3 months (e.g., cataract extraction, vitrectomy, or intravitreal anti-VEGF injection); (6) significant opacities of the refractive media that could affect the quality and assessment of fundus imaging.

Total RNA of 10 samples was extracted using TRIzol (Ambion, USA). The extracted total RNA was reverse transcribed into cDNA using the SweScript First Strand cDNA Synthesis Kit (Servicebio, Wuhan, China) following manufacturer’s protocols. RT-qPCR analysis was conducted using Servicebio’s 2× Universal Blue SYBR Green qPCR Master Mix (Wuhan, China) under standard cycling conditions. GAPDH served as the endogenous control gene for normalization of expression data. Quantitative PCR amplification was performed using the CFX Connect Real-Time PCR Detection System (Bio-Rad, USA) under the following cycling parameters: 1 min at 95℃ for pre-denaturation, 20 s at 95℃ for denaturation, 20 s at 55℃ for annealing, and 30 s at 72℃ for extension, for a total of 40 cycles. mRNA levels were quantified relative to the reference gene using the 2^−ΔΔCT^ method [31].

### Statistical Analysis

Statistical analysis was implemented in R (v 4.2.2), with between-group differences evaluated via Wilcoxon testing (significance defined as *p* < 0.05).

### Ethical approval

This research involving human subjects received ethical approval from the Institutional Review Board of The Second Affiliated Hospital of Guangxi University of Chinese Medicine (Ethical review approval number: KY2024-213, Date: 2024.02.03), with all participants providing written informed consent.

## Results

### DEGs acquisition

Analysis of the GSE221521 dataset revealed 2096 DEGs in DR samples, comprising 1567 upregulated and 529 downregulated transcripts (Fig. 2a). The GSE23371 cohort exhibited 4,021 significant DEGs in tolDCs compared to controls, with 1653 genes showing increased expression and 2368 genes demonstrating decreased expression (Fig. 2b). Analysis of the GSE52894 dataset revealed 3285 DEGs in tolDCs, consisting of 1448 upregulated and 1,837 downregulated transcripts (Fig. 2c). Analysis of the GSE182528 dataset identified 419 DEGs in tolDCs, including 270 upregulated and 149 downregulated transcripts (Fig. 2d). The GSE56017 cohort demonstrated 1346 significant DEGs in tolDCs relative to controls, with 860 genes exhibiting increased expression and 486 genes showing decreased expression (Fig. 2e). Finally, 6,267 tolDCs-related genes were identified.

### Acquisition of 51 key genes

By integrating the tolDCs-related genes with DEGs1, 221 candidate genes were identified (Fig. 3a). The identified key genes demonstrated significant enrichment in 386 GO categories and 2 major KEGG signaling pathways. For instance, GO terms included “cytoplasmic translation,” “cytosolic ribosome,” and “cadherin binding” (Fig. 3b). The KEGG pathways primarily included “Coronavirus disease-COVID-19” and “Ribosome” (Fig. 3c). In the PPI network, associations were found between 203 proteins, such as OSA3 and FASN (Fig. 3d). A total of 51 key genes were identified by using the 5 algorithms for selection (Fig. 3e).

### Identification of TFEC and CHMP2A as biomarkers

9 feature genes (feature genes 1) were identified using LASSO regression analysis (lambda.min = 0.04) (Fig. 4a). Meanwhile, 10 feature genes (feature genes 2) were identified using the Random Forest algorithm (Fig. 4b). By integrating feature genes1 and 2, 2 candidate biomarkers were identified: transcription factor EC (TFEC) and chromatin modifying protein 2 A (CHMP2A) (Fig. 4c). Cross-dataset validation in GSE221521 and GSE185011 confirmed consistent differential expression patterns, with TFEC demonstrating significant upregulation and CHMP2A showing marked downregulation in DR samples compared to controls. Therefore, TFEC and CHMP2A were classified as biomarkers (*p* < 0.05) (Fig. 4d). ROC analysis indicated that both TFEC and CHMP2A had good predictive value for DR, with AUC values of 0.770 and 0.739, respectively (Fig. 4e).

### Multiple signaling pathways in DR

GSEA identified TFEC as significantly associated with 994 biological pathways (FDR < 0.25), with prominent enrichment in the ‘Cytoplasmic Ribosomal Proteins’ pathway. CHMP2A was enriched in 748 pathways, including “tRNA Processing.” The pathways enriched by both TFEC and CHMP2A included “apoptosis,” “VEGFA-VEGFR2 signaling,” and “signaling by VEGF,” among others (Fig. 4a and b). These findings strongly implicate the VEGF-mediated signaling pathway as a critical driver of DR pathogenesis.

### Association of biomarkers with the immune microenvironment

The stacked bar plot demonstrated the percentages of 22 immune cell types in DR and control groups (Fig. 5a). The scores of 8 immune cell types showed significant differences, including M0 macrophages (*p* < 0.05) (Fig. 6b). Spearman’s correlation analysis demonstrated a significant positive association between activated NK cells and resting mast cells (ρ = 0.46, *p* < 0.001) (Fig. 6c). In addition, Spearman correlation analysis revealed that TFEC showed the strongest *negative* correlation with activated NK cells (cor = -0.49, *p* < 0.001), while CHMP2A correlated most strongly *positively* with resting mast cells (cor = 0.49, *p* < 0.001) (Fig. 6d). These results indicated that there were interactions between immune cell types and that biomarkers were involved in the progression of DR.

### Clinical experimental results

A total of 10 volunteers were enrolled in this study, including 5 patients in the DR group and 5 in the healthy control group. In the DR group, the mean age was 65.4 ± 8.6 years (2 males and 3 females), the mean glycosylated hemoglobin (HbA1c) was 8.7 ± 1.8%, and the mean duration of diabetes was 6.2 ± 5.6 years. According to the International Clinical Diabetic Retinopathy severity scale(ICDR), 2 patients had moderate non-proliferative DR (NPDR), 2 had severe NPDR, and 1 had proliferative DR (PDR). In the control group, the mean age was 64.8 ± 11.0 years (4 males and 1 female). Statistical analysis showed no significant differences in gender or age between the two groups (all *p* > 0.05), indicating comparable baseline characteristics.

RT-qPCR validation confirmed significant upregulation of TFEC (*p* < 0.01) and downregulation of CHMP2A (*p* < 0.05) in DR samples compared to controls, fully concordant with bioinformatics predictions (Fig. 7a and b). This further confirmed the reliability of the bioinformatics analysis.

## Discussion

DR is a multifactorial disease characterized by oxidative stress, chronic inflammation, and immune dysregulation. TolDCs, which suppress excessive inflammation and maintain immune homeostasis, are implicated in DR pathogenesis [32–34]. Based on GEO database bioinformatics analysis, this study identified two key genes: TFEC and CHMP2A. Multi-dataset validation and RT-qPCR confirmed that TFEC correlated negatively with activated NK cells, and CHMP2A correlated positively with resting mast cells. TFEC was significantly upregulated while CHMP2A was downregulated in the peripheral blood of DR patients. ROC analysis indicated favorable diagnostic value, suggesting that TFEC and CHMP2A may serve as potential biomarkers and therapeutic targets for DR.

 TFEC, a microphthalmia-associated transcription factor (MiT) family transcription factor, binds E-box sequences as homodimers or heterodimers to regulate target gene expression and participates in cellular processes such as survival, growth, and differentiation [35]. This study is the first to report dysregulation of TFEC in DR. GSEA showed that TFEC participates in 994 pathways, including cytoplasmic ribosomal protein synthesis. Immune infiltration analysis revealed a disordered retinal immune microenvironment in DR, with TFEC exhibiting the strongest negative correlation with activated NK cells. NK cells are critical innate immune cells that recognize and eliminate abnormal cells (e.g., tumor cells, virus-infected cells, senescent cells), thereby limiting tissue injury. They also secrete cytokines and chemokines such as IL-10 and TGF-β to modulate innate and adaptive immunity, inhibit overactivated T cells and DCs, prevent tissue damage caused by excessive immune activation, and maintain immune homeostasis [36–37]. Emerging evidence has shown reciprocal crosstalk between NK cells and neutrophils. Neutrophils activate NK cells, which further induce the formation of neutrophil extracellular traps (NETs) to mitigate retinal vascular leakage and inhibit pathological neovascularization. Functional NK cells are indispensable for sustaining retinal homeostasis [38]. In addition, evidence indicates that diabetes leads to NK cell subset imbalance and dysfunction, characterized by decreased mature highly cytotoxic NK cells in peripheral blood, accompanied by impaired cytotoxicity and cytokine secretion. NK cell dysfunction further aggravates immune imbalance through the IL-2/Tregs axis, accelerating diabetes progression [39–40]. Our clinical RT-qPCR validation confirmed that TFEC was significantly upregulated in the peripheral blood of DR patients, which may indicate a marked reduction in activated NK cells. Based on these findings, TFEC may serve as a key mediator of NK cell dysfunction in diabetes. TFEC impairs retinal immune surveillance and damaged cell clearance, further inducing inflammation, vascular leakage and pathological neovascularization to drive DR pathogenesis.

CHMP2A is located on human chromosome 19q13.43. As a CHMP family member, it is a core component of the ESCRT-III complex, which mediates endosomal sorting [41]. Non-healing wounds or diabetic ulcers are serious complications of diabetes. Research reveals that CHMP2A protein levels are altered at wound sites in diabetic obese individuals as early as day 1 post-injury, which is associated with a failure to enter the proliferative phase of healing [42]. CHMP2A regulates endoplasmic reticulum (ER) stress responses, and its expression is altered under ER stress to modulate intracellular protein sorting, trafficking, and vesicle formation, thereby promoting cellular adaptation. ER stress plays a critical role in diabetes, and CHMP2A dysfunction may compromise the ER stress response, disrupt normal cellular functions, and contribute to disease progression [43]. GSEA in this study showed that CHMP2A was enriched in 748 pathways, including tRNA processing, and exhibited the strongest positive correlation with resting mast cells. Mast cells are widely distributed innate immune cells commonly located adjacent to blood vessels and nerves. Under hyperglycemia, oxidative stress, and inflammatory stimulation, resting mast cells markedly decrease and convert to an activated phenotype. Activated mast cells release histamine, tryptase, VEGF, and pro-inflammatory cytokines, which exacerbate vascular leakage, endothelial injury, and pathological neovascularization, directly damage myelin sheaths of nerve fibers, and recruit more immune cells to trigger an inflammatory storm [44-45]. Clinical RT-qPCR confirmed that CHMP2A was significantly downregulated in DR patients, which may indicate a marked reduction in resting mast cells in peripheral blood. We thus speculate that resting mast cell depletion and homeostatic imbalance may contribute to DR, and that CHMP2A preserves retinal immune homeostasis and blood-retinal barrier integrity by maintaining mast cell quiescence and limiting excessive inflammation.

We further explored the roles of TFEC and CHMP2A in DR. GSEA revealed overlapping enrichment of the two genes in core DR-associated pathways, including apoptosis and VEGF signaling (Fig. 4A and B). Accumulated evidence also confirms that serum VEGF level is closely linked to DR severity, serving as a reliable biomarker [46]. Consistently, our pathway analysis indicated that TFEC and CHMP2A coordinately regulate VEGF-related signaling and apoptotic pathways, thereby synergistically driving DR pathogenesis.

Beyond regulating VEGF and apoptotic pathways, TFEC and CHMP2A may also contribute to DR progression by modulating the local immune microenvironment. Our immune infiltration analysis identified eight differentially abundant immune cell types in DR, including CD8^+^ T cells, monocytes, and M1 macrophages. The proinflammatory DR microenvironment drives M1 macrophage polarization and NF-κ B activation, promoting inflammatory cytokine release and exacerbating retinal damage [47–49]. Peripheral blood M1 macrophage markers may enable early DR diagnosis and treatment planning.

In summary, we identified two tolDCs-associated biomarkers (TFEC and CHMP2A) via bioinformatics analysis, and investigated their biological functions and associations with the immune microenvironment. This study is the first to propose that the TFEC-activated NK cell axis and CHMP2A-resting mast cell axis together form a key regulatory network underlying immune microenvironmental imbalance in DR. These findings were subsequently validated in clinical specimens. Hyperglycemia upregulates TFEC and downregulates CHMP2A, suppressing NK cell surveillance and disrupting mast cell homeostasis, thereby aggravating retinal inflammation and vascular damage to promote DR pathogenesis. This study provides new immunological insights into DR pathogenesis and potential targets for its early diagnosis and therapy. Limitations include small sample size, limited mechanistic insight, and insufficient functional validation; future studies should expand cohorts and perform in vitro/in vivo experiments.

## Data Availability

The datasets supporting this study are available in public repositories.
